# Evaluation of Antioxidant Performance in Chromium Oxidation Prevention

**DOI:** 10.3390/ma18081858

**Published:** 2025-04-18

**Authors:** Omar Salmi, Giulia Laudisa, Filippo Rossi, Maurizio Masi

**Affiliations:** Dipartimento di Chimica, Materiali e Ingegneria Chimica “Giulio Natta”, Politecnico di Milano, Piazza Leonardo da Vinci 32, 20133 Milano, Italy; giulia.laudisa@polimi.it (G.L.); filippo.rossi@polimi.it (F.R.); maurizio.masi@polimi.it (M.M.)

**Keywords:** antioxidants, chromium tanning, leather

## Abstract

The tanning of hides is a practice deeply rooted in ancient times, but in the early 20th century, transitioning to an industrial model of leather and hide production, chrome tanning expanded globally due to its practicality, quality, and versatility. However, in recent decades, there has been a growing attention paid to the potential oxidation of the free chromium present in tanned leather, which could transform from an unharmful trivalent status into its carcinogenic hexavalent status. This phenomenon occurs in a very small fraction of hides, which is yet sufficient to exhibit significant activity. Hence, there is an evident need to explore further alternatives that allow avoiding oxidation. In this work, the performance of a sequence of selected antioxidants is evaluated in different oxidation conditions: simple stirring, UV–Vis–IR irradiation, and heating up to 75 °C. The official diphenylcarbazide–UV tool norm was used to quantify the hexavalent chromium amount. The results underline the effectiveness of 2,6-di-tert-butylphenol, and that its greatest preventative method of addition during the tanning process is together with the tanning agent. This approach will pave the way for researching alternative concepts and exploring perspectives to inhibit chromium issues.

## 1. Introduction

Leather has proven to be one of the fastest-growing segments within the global market for personal luxury goods and is expected to grow at rates above the market average [1]. The improvement stems from increasing consumer incomes and enhanced living standards, which have resulted in a greater demand for leather products [2]. Leather industries hold economic significance as they enhance the value of raw hides that, after the slaughterhouse phase, are considered almost totally a waste. Tanneries emphasize these useful animal resources, ensuring that the less-used parts of the animal do not go to waste [3].

From an industrial point of view, the transition process from raw hides to a refined product is called tanning and encompasses several steps. In its entirety, irreversible cross-links are established between polypeptide chains in this process, which constitute the smallest structural units of leather collagen fibers [4]. Historically, until the 19th century, vegetal tannins represented the prevalent method for stabilizing collagen, yielding a harder type of leather. However, in the late 19th century, chromium was introduced, offering a faster, cost-effective, and versatile product, suitable for many different industries [5]. The chemicals employed in tanning may vary according to the tanning mode, and chromium tanning has continued to be the widest employed method [6]. In particular, a Cr(III) salt is used, representing the most stable oxidation state of chromium [7] and characterized by the ability to make stable complexes that strongly stabilize collagen with coordinative bonds [8].

However, the unlikely but possible oxidation of trivalent chromium may lead to the observation of the hexavalent compound. Specific occupational risks are associated with the hexavalent metal, which is heavily problematic [9], not only causing direct tissue damage, but also containing carcinogenic and mutagenic potential [10]. Besides the lung and the nose mucous tissue, the kidney is the main target organ after uptake of Cr(VI). In more detail, after oral and/or dermal exposure, the different absorption rates of Cr(III) and Cr(VI) compounds account for specific respective profiles of toxicity [11], where Cr(VI) compounds easily penetrate a cell membrane in the state of chromate anions [12]. Additionally, in the case of the environment, chromium issues cannot be avoided. When a soil contains a large amount of soluble and biologically assimilable forms of chromium, the effects of soil pollution from the metal ion are observed [13]. The soluble ionic status of Cr(VI) formed in or added to soils or natural waters will persist indefinitely unless chromate is removed by leaching, adsorption, precipitation, uptake by living cells, or by reduction to the trivalent form [14]. All these reasons are behind the focus on the possible formation of Cr(VI) [15].

Fatliquoring is an additional key step of tanning for enhancing leather quality, specifically by improving softness, wear resistance, tear resistance, and porosity [16]. The fatliquoring agents’ role is to prevent the collapse of collagen fibers caused by the removal of water during the drying process by working as lubricants [17]. The chemicals used in the operation derive from four primary sources: fish, petroleum, vegetable, or animal origins. Some of the miscellaneous extracted products are rich in fatty acids, often with a significant proportion of unsaturated fatty acids [18]. The last mentioned ones, represented by fish oils, may, after photo-aging with UV light or thermal treatment, lead to the oxidation of Cr(III) [19]. This explains why, ever since testing began in tannery wastewaters, influenced by further external factors, it has been possible to find Cr(VI), even if in lower amounts thanks to the humidity effect. In fact, storage of fatliquored leather at a relative humidity above 35% may result in an increase of probable Cr(VI) formation [20]. These oxidative agents have physicochemical properties that can introduce hydroperoxides, free radicals, and other reactive species to double bonds, thereby inducing the oxidation of trivalent chromium to hexavalent chromium. This oxidation primarily affects the residual free metal within the tanned leather, which is more prone to oxidation compared to complexed metal.

In this context, there is an urgent need for solutions to mitigate or inhibit this oxidation process [21]. Other possibilities were studied in detail, with particular attention paid to tara tannin and ascorbic acid. Since no satisfying effects were presented in tanneries, the purpose of the present work is to find an optimum method that reasonably reduces chromium oxidation in tanning without any side effects on the final leather (typical of vegetal tannins). The performance of the antioxidant and the sequence of additions to be made to ensure the lowest amount of the oxidation product have been investigated. A selection of antioxidants has been made on the basis of a previous study [22], by choosing antioxidants among the most common of those already industrially used and a commercialized product, and their performance has been evaluated.

The study in question is a groundbreaking discovery that improves the tanning process, enabling the dual action of ensuring safety while simultaneously guaranteeing high product quality. The proposed comparison allows us to understand the effects of the chosen antioxidants on the formation of hexavalent chromium.

## 2. Materials and Methods

### 2.1. Materials

The compositions of the listed agents are based on those declared in the safety sheets.

The commercial fatliquoring agent used is Riveroil TIS, composed of sulphonate esters, polyglycol ethers, fish oil derivates, and alkanediols (supplied by River Chimica Industriale S.p.A., San Miniato (PI), Italy).

The tanning agent used is Cromo FD, composed of Cr(OH)SO_4_ + Na_2_SO_4_ > 75% (supplied by River Chimica Industriale S.p.A., San Miniato (PI), Italy).

The antioxidant screening involved the following chemicals: 2,6-di-tert-butylphenol (C_14_H_22_O, MW = 206.32 g/mol, CAS: 128-39-2, Fluka, Buchs, Switzerland); DL-α-tocopherol (C_29_H_50_O_2_, MW = 430.71 g/mol, CAS: 10191-41-0, Tokyo Chemical Industry Co., Tokyo, Japan); 2,2,6,6-tetramethylpiperidinoxy free radical, TEMPO (C_9_H_18_NO, MW = 156.25 g/mol, CAS: 2564-83-2, Fluorochem Ltd., Hadfield, UK); 2,5-di-tert-butylhydroquinone (C_14_H_13_O_2_, MW = 222.32 g/mol, CAS: 88-58-4, Sigma-Aldrich, St. Louis, MO, USA); and a declared antioxidant product available on the market and its active principle.

The solvents used for extractions are the following: ethyl acetate, EtOAc (C_4_H_8_O_2_, MW = 88.1 g/mol, CAS: 141-78-6, Sigma-Aldrich, St. Louis, MO, USA); dichloromethane, DCM (CH_2_Cl_2_, MW = 84.93 g/mol, CAS: 75-09-2, Sigma-Aldrich, St. Louis, MO, USA); and formic acid (CH_2_O_2_, MW = 46.03 g/mol, CAS: 64-18-6, Sigma-Aldrich, St. Louis, MO, USA).

Additionally, Freiberg hide powder (FILK, Freiberg Institute, Freiberg, Germany) was used.

To perform the Cr(VI) quantification in water and leather (EN ISO 17075-1), the following were used: potassium hydrogen phosphate (K_2_HPO_4_, MW = 174.18 g/mol, CAS: 7758-11-4, Sigma-Aldrich, St. Louis, MO, USA); 1,5-Diphenylcarbazide (DPC) (C_13_H_14_N_4_O, MW = 242.28 g/mol, CAS: 140-22-7, Sigma-Aldrich, St. Louis, MO, USA); sulfuric acid (H_2_SO_4_, MW = 98.08 g/mol, CAS: 7664-93-9, Sigma-Aldrich, St. Louis, MO, USA); phosphoric acid (H_3_PO_4_, MW = 97.99 g/mol, CAS: 7664-38-2, Sigma-Aldrich, St. Louis, MO, USA); and potassium dichromate (K_2_Cr_2_O_7_, MW = 294.19 g/mol, CAS: 7778-50-9, Sigma-Aldrich, St. Louis, MO, USA).

### 2.2. Methods

The study is divided into two steps, following the same principle: Cr(VI) becomes quantifiable because diphenylcarbazide (DPC) is oxidized by the hexavalent chromium and becomes diphenylcarbazone (DPCA), detectable on the UV spectrophotometer.

#### 2.2.1. Antioxidant Efficacy in Tannery Wastewater

In a beaker, a primary solution of 60 mL reproduces the generic conditions of the substance combination in finished leather (percentages are based on the total volume). The detailed recipe is made up of 44.9% water, 24.9% Cromo FD, and 29.9% of Riveroil TIS [23]. The tests are conducted in a 6-well plate, and each has a volume of 6 mL. The antioxidant is added directly in the wells at 0.3% of the single well volume. The plates are kept in motion by magnetic stirrers at approximately 300 rpm. Three different oxidative conditions were tested: simple stirring for 5 days at room temperature, UV–Vis–IR irradiation for 3 days [24] at room temperature (25 °C), and heating at 75 °C for 2 days. After processing, the 6 mL are diluted with water in a 50 mL flask. To carry out prior isolation of interfering substances (solid powder/antioxidant/fattening agents), a Buchner filtration is performed for each, and 1 mL of filtrate is diluted with water in 25 mL volumetric flasks. Then, liquid–liquid extraction is done using a separating funnel, using EtOAc and DCM with a maximum of three washes per solvent. At this point, the solution is ready for the normative reference from the CNR-IRSA Italian national institute: a spectrophotometric determination of Cr(VI) using diphenylcarbazide (DPC) directly in a water solution [25].

#### 2.2.2. Antioxidant Efficacy with Hide Powder

This research involves antioxidant experiments made on hide powder, with the normative reference following EN ISO 17075-1:2017 [26]. The weight of the experimental formulation is measured on the basis of the wet hide powder. The best-performing antioxidant selected in the first step is tested. The procedure consists of the following steps: 1 g of hide powder is wet with a few mL of water until it reaches the weight of 7.8 g; then, on the basis of the wet powder weight, 40% *w*/*w* of acidified water (water + formic acid) at pH 2.5 treats the hide [27]; after 24 h, 8% *w*/*w* of Cromo FD is added [28]; and after another 24 h, 9% *w*/*w* of Riveroil TIS is finally added to the solution. Each antioxidant is added at 1% *w*/*w* of the fatliquoring agent quantity, and for each one, three different solutions have been prepared, differentiated by the order in which the antioxidant is added. They are addressed, respectively, with the letters Q, W, and E. In solution Q, the antioxidant is added together with the fatliquoring; in solution W, it is added right after the acidic water (simulates pickling [29]); and in solution E, it is added right after the chromium addition. In this way, the extraction is followed by the specific normative steps.

### 2.3. Instruments

The hexavalent chromium quantification was conducted in both cases using UV spectrometry. The instrument used is a V-600 Series UV–Vis spectrophotometer from JASCO (Cremella (LC), Italy). A 540 nm wavelength was selected [30], with a range between 0.1 and 1 mg/L Cr(VI). The quantity of Cr(VI) in the liquid sample was calculated using two different calibration curves, according to the different normative references followed. In the first method (CNR-IRSA), cuvettes with a 1 cm optical path were used, while for the second procedure (ISO), the optical path is 5 cm, which is favorable for sensitive incrementation. The pH measurements were performed using a lab pH meter (HI98191, Hanna Instruments, Padova, Italy) with a standard error of 0.01.

## 3. Results and Discussion

The purpose of this work was to find the best antioxidant able to prevent the oxidation of trivalent chromium in tannery wastewaters, and then directly on leather. To this end, the quantities of each compound were chosen to reproduce the worst case in the industrial process: the excess of a sensible (but useful) recipe. To facilitate the reproducibility of the results, a recognized standard collagen matrix was used: Freiberg hide powder. Below are the steps that our research conducted to evaluate the performance of the antioxidants. The UV principle remains the same: diphenylcarbazide (DPC) is oxidized by the hexavalent chromium and becomes diphenylcarbazone (DPCA) [31], making a complex with the oxidized metal, which is detectable on the UV spectrophotometer.

### 3.1. Experimental Set-Up

**Figure 1:**
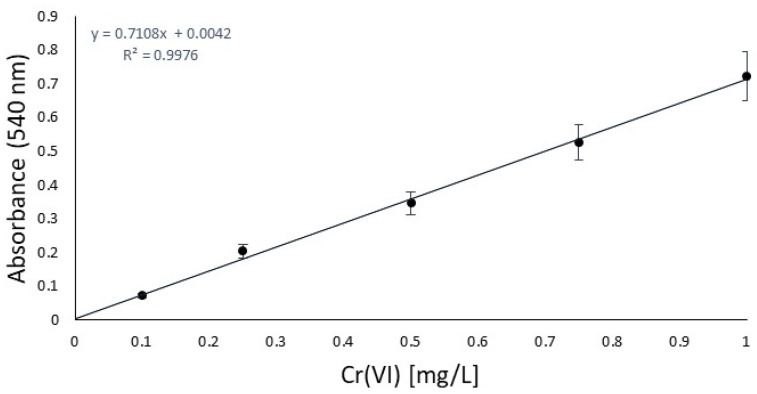
Calibration curve according to the CNR-IRSA normative reference [35].

First of all, in order to focus on the antioxidant performance, a chromium salt as the tanning agent and a fatliquoring agent had to be chosen and kept unchanged for all the experiments to ensure that the results would be effectively compared. From a previous study [22], to represent the most sensible conditions for oxidation, Cromo FD and Riveroil TIS were selected, respectively. On the same basis, the series of antioxidants to be checked were decided, focusing our investigation on 2,6-di-tert-butylphenol, TEMPO, and tocopherol, which had already been tested in the previous work only under simple stirring conditions. To this list, a declared antioxidant product available on the market, its active principle, and 2,5-di-tert-butylhydroquinone were added. To control oxidation, the idea was to use the same sequence of steps as the industrial tanning process and quantities of chromium that simulate the real conditions in tanneries in the worst case, without the basification that enhances the stabilization of chromium complexes [32]. At this last point, the availability of free chromium is increased, directly related to the oxidation sensibility increasing. The decision to test three different operating conditions was due to the hexavalent chromium expected. The UV irradiation and temperature should facilitate the oxidation of trivalent chromium [33], and so represent the worst conditions for the analysis. After the oxidation time, to carry out prior isolation of interfering substances (antioxidant/fatliquoring agents), filtration was performed for each. Then, liquid–liquid extraction was performed to avoid the risk of running into an auxiliary reaction of DPC or measurement mistakes. In order to validate the procedure of Cr(VI) quantification, a blank study in the absence of an antioxidant was done [34]. Before the analysis, the calibration curve was obtained between 0.1 and 1 mg/L, as reported in Figure 1, and according to the normative reference [35]. This was essential for the quantification of Cr(VI) by UV absorption in the next step.

### 3.2. UV Analysis

**Figure 2:**
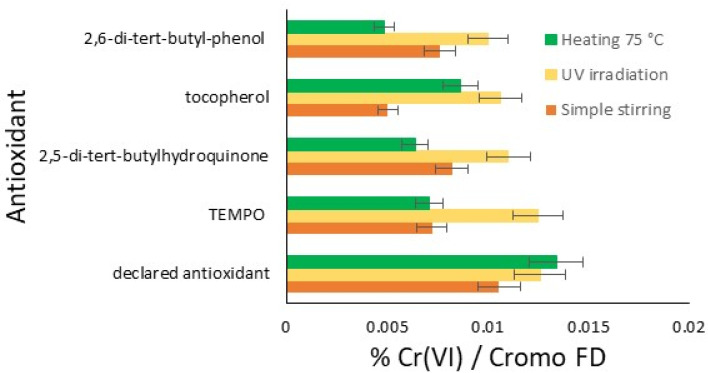
Summarizing the results: comparison of the efficiency of the antioxidant on the amount of Cr(VI) formed from Cr(III) (%): simple stirring (orange); UV irradiation (yellow); and heating at 75 °C (green).

In the graph in Figure 2, the results of this initial comparative study are reported for the three conditions tested: stirring for five days, UV irradiation for three days, and the thermal effect at 75 °C for two days. Therefore, the amount of hexavalent chromium for each antioxidant under each oxidation condition is possible to see in the bar chart. Each trial resulted in an amount of chromium significantly lower than the blank, which was completely out of scale, resulting in 97.17% of hexavalent chromium with Cromo FD. The results are classified from the best to the lowest effect.

In general, UV irradiation tends to produce more Cr(VI) compared to heating and stirring, except in the case of the declared commercial antioxidant, where heating produces slightly more Cr(VI) than UV irradiation. This result represents a first important limitation of the studied antioxidants. With regard to heating, better antioxidant protective activity is observed with all the compounds, and some sensitiveness is only found in the cases of the declared antioxidant and tocopherol. Finally, the easiest stirring represents the best protective condition for the limitation of Cr(VI) development, with the exception of the declared antioxidant. The radical molecule TEMPO, now widely used in chemical environments and the plastics industry, which is of synthetic origin, exhibits interesting antioxidant activity, sensitive only to UV. Notably, an antioxidant effective under irradiation does not necessarily exhibit the same characteristics in tests conducted at elevated temperatures. In the tests at 75 °C, 2,5-di-tert-butylhydroquinone, TEMPO, and 2,6-di-tert-butylphenol show much greater efficacy. The declared antioxidant product available on the market, on the other hand, has relatively comparable activities across different conditions.

**Figure 3:**
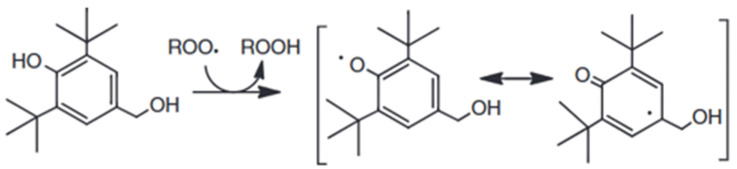
Stabilization of hindered phenols by resonance.

The indicated performance should be related to the chemical structure of these molecules. 2,6-di-tert-butylphenol and 2,5-di-tert-butylhydroquinone are two hindered phenols, characterized by a strong tendency to be oxidized and to remain stable for a long time [36]. In fact, their hindered structure permits the shear of the inducted charge along all the C-C bonds. The difference between them is the presence of one more hydroxide group and the lesser hindering of the tert-butyl group. This is why 2,6-di-tert-butylphenol shows better behavior [37]. A similar explanation is represented in Figure 3 [36].

In the case of tocopherol, the mechanism is different, and not comparable with the other chemicals. Its aromatic hydroxide is oxidized, becoming easily stabilized by the many aliphatic carbons in the chemical structure [38]. Additionally, in harder oxidative conditions (oxidating agent, heating, and irradiation), its behavior cannot be explained [39]. In the case of TEMPO, there is another method of preventing oxidation, and its power is related to its capacity to regenerate itself using a redox mechanism [40]. The possible problem that caused the strong oxidation under UV rays is related to its reduction. In fact, in order to regenerate itself, TEMPO must oxidize other compounds and oxidize fatty acids, eventually leading to the same oxidative behavior that it initially lacks; if chromium is oxidized by itself, it is even more problematic.

### 3.3. UV Analysis on Hide Powder

**Figure 4:**
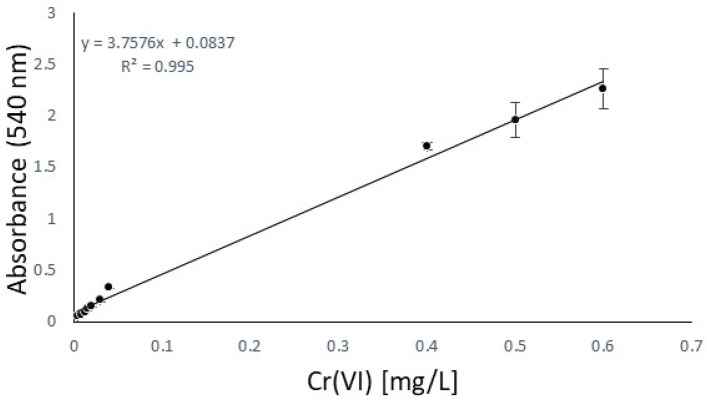
Calibration curve: Cr(VI) in the extraction solution based on ISO 17075-1.

The hide powder is involved in this phase, where two different aspects are to be evaluated. First of all, this phase aims to find the most effective antioxidant, and secondly, it aims to assess whether adding it in the phase of acidification, tanning, or fatliquoring will produce the maximum effect on the inhibition of hexavalent chromium formation. From the previous step, further screening was conducted, selecting four antioxidants to be tested directly on the hide powder: 2,5-di-tert-butylhydroquinone, tocopherol, 2,6-di-tert-butyl-phenol, and the active principle of the declared antioxidant product available on the market. The calibration curve, using potassium dichromate according to the normative reference EN ISO 17075-1, is reported in Figure 4 [26], and permits the analytical quantification of Cr(VI) directly by UV absorption. In particular, the method is described in the relative ISO to be suitable to quantify the Cr(VI) content in leathers down to 3 mg/kg. Due to the low concentration of Cr(VI) extracted from leather after the standard procedure detectable with a UV spectrophotometer, cuvettes with a 5 cm cell optical path were chosen, which have a well-known application in this type of analysis to increase absorbance proportional to the optical path length [41]. Furthermore, for an improvement of UV quality, a filtration was done after the oxidation procedure to avoid the risk of running into an auxiliary reaction of DPC or measurement mistakes (as scattering in UV analysis) [42]. Blank trials were also carried out to evaluate the presence of Cr(VI) in the absence of an antioxidant.

Three different conditions were also tested here, as in the previous step: simple stirring for 5 days, UV–Vis–IR irradiation for 3 days, and heating at 75 °C for 2 days.

**Figure 5:**
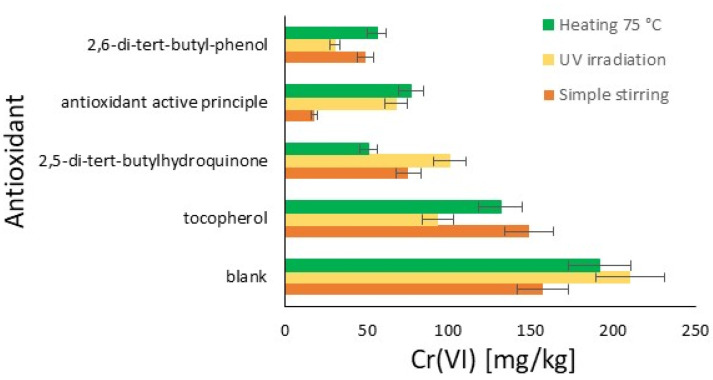
Comparison of antioxidants’ effect on Cr(III) oxidation to Cr(VI) in hide powder with respect to a blank in different conditions: simple stirring (orange); UV irradiation (yellow); and heating at 75 °C (green).

The graph in Figure 5 shows the quantity of Cr(VI) in the hide powder under various conditions, with and without the use of different antioxidants. The quantity of Cr(VI) is expressed in mg/kg on the x-axis, while the tested antioxidants are listed on the y-axis. The absence of antioxidants leads to the highest quantities of Cr(VI), confirming that exposure to UV light, agitation, and heating facilitates the formation of Cr(VI) in the absence of antioxidant protection. 2,5-di-tert-butylhydroquinone significantly reduces the formation of Cr(VI) compared to the blank, with the highest efficacy observed during heating at 75 °C. 2,6-di-tert-butylphenol proves to be the most effective antioxidant, reducing the formation of Cr(VI) to very low levels under all tested conditions, with success under UV light. The commercial antioxidant proves to be very effective during agitation but shows reduced efficacy under UV light and heating compared to 2,6-di-tert-butylphenol. Tocopherol is the least effective among the tested antioxidants, especially under conditions of agitation and heating, where it shows high quantities of Cr(VI).

The aim here is to evaluate how the characteristics change between Figure 2 and Figure 5 (with and without hide) in terms of their efficiency classification. 2,6-di-tert-butylphenol remains the substance with the most effectful characteristics, demonstrating that its structure provides important prevention for all the oxidizing phenomena [43]. Similar behavior between the experiments with and without hide powder is demonstrated also in the case of 2,5-di-tert-butylhydroquinone. The active principle of the commercial antioxidant’s behavior differs a lot from the formulated one, and demonstrates strong potential in application. The presence of additives in the formulation limits this effect, but otherwise may increase penetration into the raw skin and/or limit the interaction with other chemicals or effects on the organoleptic properties. A completely different behavior in powder hide was found in the case of tocopherol. In the case of the collagen presence, the antioxidant cannot provide the same effectiveness, representing a limit that could be due to some possibilities such as the different concentration or direct differences in the extraction. In fact, in the first case, the possibility should be due to the lower efficiency in leather at this concentration, as declared in the literature [44]. In the case of the second method, the hexavalent chromium is extracted by a solution of K_2_HPO_4_⋅3H_2_O and H_3_PO_4_ [45], so the high pH in this passage and the presence of a transition metal could limit the effect of tocopherol according to its hydrogen-donating ability (HAT) [46].

**Figure 6:**
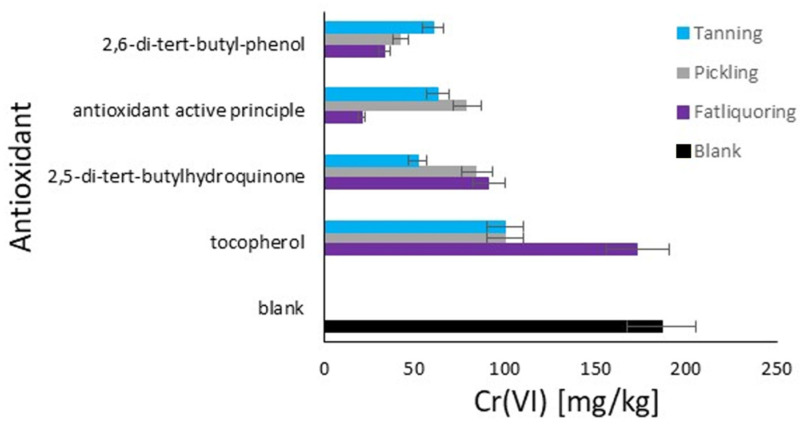
Comparison of the antioxidants’ effect on Cr(III) oxidation to Cr(VI) in hide powder with different moments of addition: light blue (E)—tanning; grey (W)—pickling; and purple (Q)—fat liquoring. Black (B)—blank, no antioxidant.

The graph in Figure 6 displays the quantity of hexavalent chromium in the hide powder based on the order of antioxidant additions. The quantities of Cr(VI) are expressed in mg/kg on the x-axis, while the tested antioxidants are listed on the y-axis. The different modes of antioxidant addition are represented by four colors, as described. In the blank, the reference value is 186 mg/kg of Cr(VI), representing the highest quantity, which resulted from the average value of the blank study of the data previously shown. This highlights, once again, the necessity of the antioxidant. 2,5-di-tert-butylhydroquinone shows the highest efficacy when added together with chromium (E), with a quantity of approximately 51 mg/kg of Cr(VI). When added before tanning (W) or together with fatliquor (Q), it is less effective. For 2,6-di-tert-butylphenol, when added together with fatliquor (Q), it is more effective, by a quantity of approximately 33 mg/kg of Cr(VI). Its efficacy decreases when added before tanning (W) and together with chromium (E). The active principle of the formulated product demonstrates a maximum efficacy when added together with fatliquor (Q), with only 21 mg/kg of Cr(VI). It is less effective when added together with chromium (E) and before tanning (W). Tocopherol appears to be the least effective among the tested antioxidants, with elevated quantities of Cr(VI) in all addition modes, especially when added together with fatliquor (Q), with approximately 173 mg/kg of Cr(VI).

The trend remains the same as in Figure 5 in terms of antioxidant activity, and an interest point arises about the addition with fatliquor for tocopherol, which demonstrates the highest problem with its use. The reason for this behavior should be related to its possible immediate deactivation by the direct mixing of sensible oxidative substances with tocopherol [47]. The rapid antioxidant reaction caused by its important affinity with fats represents a limit respective to the other agents [48], as it results in a short activity period. The study of the effects of additions during the pickling phase was decided to stress the system with an acidic pH, which improved or limited the antioxidant effects for the same reasons explained in the discussion of Figure 5, as the classifications remain the same. Finally, in the case of the addition with chromium, 2,5-di-tert-butylhydroquinone is the best for its affinity with chromium and lower affinity with the fatliquoring phase, due to its hydroxy groups that ensure a fundamental polarity respective to the other analyzed molecules [49].

The addition of antioxidants with tanning or fatliquoring agents is a practice already known and used in tanneries [50]; however, this study suggests which agents could explain the best effect. From another point of view, these results could indicate possible strategies to also obtain an additive effect, for example, by using the commercial antioxidant active principle with the fatliquoring agents, and 2,5-di-tert-butylhydroquinone with the tanning agent.

## 4. Conclusions

In terms of overall efficacy, 2,6-di-tert-butylphenol emerges as the most effective antioxidant in all tested conditions, showing the lowest levels of Cr(VI) in the hide powder and important safe activity in tannery wastewater. From the perspective of oxidation under specific conditions, the active principle is very effective under UV light, but less crucial during agitation and heating. The antioxidant with variable performance is 2,5-di-tert-butylhydroquinone, which shows good efficacy, particularly under heating. The least effective antioxidant is tocopherol, which, while reducing the Cr(VI) formation compared to the blank, is not as effective as the other antioxidants. The data suggest that the optimal choice of antioxidant depends on the specific conditions to which the leather material is exposed, according to a chemical configuration that improves stability. Furthermore, the best method of addition appears to be together with the chromium (E procedure). Regarding the use of antioxidants in combination with fatliquors, the least effective combination exhibits high quantities of Cr(VI), possibly due to a direct and rapid antioxidant deactivation effect. These results suggest that the optimal choice of antioxidant and the addition method can significantly reduce the formation of Cr(VI) in hide powder, thus improving the safety and quality of the final product.

## Data Availability

The original contributions presented in the study are included in the article, further inquiries can be directed to the corresponding author.

## Abbreviations

The following abbreviations are used in this manuscript:

TEMPO	Tetramethylpiperidinoxy free radical
EtOAc	Ethyl acetate
DPC	1,5-Diphenylcarbazide
DPCA	Diphenylcarbazone
